# Sex- and age‐dependent alterations of splenic immune cell profile and NK cell phenotypes and function in C57BL/6J mice

**DOI:** 10.1186/s12979-021-00214-3

**Published:** 2021-01-08

**Authors:** Kelly B. Menees, Rachael H. Earls, Jaegwon Chung, Janna Jernigan, Nikolay M. Filipov, Jessica M. Carpenter, Jae-Kyung Lee

**Affiliations:** Department of Physiology and Pharmacology, University of Georgia, College of Veterinary Medicine, 501 D.W. Brooks Drive, Athens, GA 30602 USA

**Keywords:** Ageing, Sex, Natural killer cell, Immunosenescence, α-synuclein

## Abstract

**Background:**

Physiological homeostasis decline, immunosenescence, and increased risk for multiple diseases, including neurodegeneration, are all hallmarks of ageing. Importantly, it is known that the ageing process is sex-biased. For example, there are sex differences in predisposition for multiple age-related diseases, including neurodegenerative and autoimmune diseases. However, sex differences in age-associated immune phenotypes are not clearly understood.

**Results:**

Here, we examined the effects of age on immune cell phenotypes in both sexes of C57BL/6J mice with a particular focus on NK cells. We found female-specific spleen weight increases with age and concordant reduction in the number of splenocytes per gram of spleen weight compared to young females. To evaluate sex- and age-associated changes in splenic immune cell composition, we performed flow cytometry analysis. In male mice, we observed an age-associated reduction in the frequencies of monocytes and NK cells; female mice displayed a reduction in B cells, NK cells, and CD8 + T cells and increased frequency of monocytes and neutrophils with age. We then performed a whole blood stimulation assay and multiplex analyses of plasma cytokines and observed age- and sex-specific differences in immune cell reactivity and basal circulating cytokine concentrations. As we have previously illustrated a potential role of NK cells in Parkinson’s disease, an age-related neurodegenerative disease, we further analyzed age-associated changes in NK cell phenotypes and function. There were distinct differences between the sexes in age-associated changes in the expression of NK cell receptors, IFN-γ production, and impairment of α-synuclein endocytosis.

**Conclusions:**

This study demonstrates sex- and age-specific alterations in splenic lymphocyte composition, circulating cytokine/chemokine profiles, and NK cell phenotype and effector functions. Our data provide evidence that age-related physiological perturbations differ between the sexes which may help elucidate sex differences in age-related diseases, including neurodegenerative diseases, particularly Parkinson’s disease, where immune dysfunction is implicated in their etiology.

## Background

Ageing is associated with the loss of physiological homeostasis, impaired biological function, and increased vulnerability to death [1]. According to the most recent World Population Prospects report, 1 in 6 people in the world will be over the age of 65 by the year 2050 [2]. Additionally, the population aged 80 or older will triple from 2019 to 2050 [2]. Therefore, it is critical to characterize age-related physiological perturbations to better serve the increasing ageing population. Although ageing is a conserved process across species, multiple aspects of the ageing process within the same species are sex-biased [3]. For example, within the ageing immune system, it is reported that while similar changes may take place in both sexes, overall rates of these immune system changes differ between the sexes (reviewed in [4]). Despite these differences, most studies have overwhelmingly favored the use of males, without consideration of sexually dimorphic effects on disease prevalence, intervention efficacy, and outcomes. Several age-related neurodegenerative diseases have sex-associated differences in prevalence including Parkinson’s disease (PD) (higher prevalence in males) [5], multiple sclerosis (higher prevalence in females) [6], and Alzheimer’s disease (higher prevalence in females) [7]. While the mechanisms underlying sex differences in these diseases are not well understood, a strong link exists between different immune system states, especially inflammation, of males and females and their propensity to develop certain diseases [8]. Inflammageing, or the reshaping of cytokine expression patterns with a progressive tendency toward a pro-inflammatory phenotype, is a characteristic feature of both ageing and age-related diseases [9].

Throughout ageing, an intricate process of reorganizational changes, collectively termed immunosenescence, occurs in the immune system [10, 11]. The major facets of immunosenescence include persistent low-grade inflammation (inflammageing), decreased abilities to fight infections or cancers, impaired ability to efficiently respond to new antigen, increased incidence of autoimmunity, and impaired wound repair [12, 13]. The process of immunosenescence affects both the immune cell repertoire and their intrinsic functional capacity. A recent study reported age-dependent reductions in CD4 + T cells, CD8 + T cells, and B cells and an increase in natural killer (NK) cells in peripheral blood across the human lifespan [14]. These alterations in immune cell composition may lead to functional deficiencies such as augmented reactive oxygen species (ROS) production (CD8 + T cells), auto-antigen specificity (B cells), or decreased cytotoxicity (NK cells) [15]. Mice display a similar immunosenescent phenotype as a reduction in the CD4 + T cell population in ageing mice has also been associated with defective cytotoxic CD8 + T cell responses leading to increased infection and inflammation [16, 17]. Furthermore, previous studies revealed an age-dependent reduction of NK cells in mouse spleen, liver, lung, and blood [18]. It has also been established that there are phenotypic and functional alterations of NK cells during healthy ageing [19]. There are limited reports that both innate and adaptive immune system responses differ with age between the sexes (reviewed in [20]). For example, it has been illustrated that NK cells from aged females are more effective in cancer immunosurveillance compared to those of aged males [20, 21]. Furthermore, a recent study revealed that ageing may impact B cells oppositely in males and females [22]. Márquez et al. showed that B cell-specific loci/genes were inactivated with age in males, but activated in females which may influence sex-differences in humoral immunity [22]. However, sex differences in immunosenescence are not clearly understood and studies investigating these differences are scarce.

NK cells are innate lymphocytes with pleiotropic functions including cytotoxicity through the production of perforin, granzyme, and interferon-gamma (IFN-γ) upon interaction with malignant cells [23], antitumor activity [24], and modulating inflammation through interactions with adaptive immune cell counterparts [25–27]. Age-related changes in NK cell frequency and function may increase susceptibility to viral infections, decrease anti-microbial immunity, and alter modulation of inflammation. The general consensus in the field is that NK cell cytotoxic capacity declines with age [28–30] potentially due to decreased expression of perforin [31] and dysregulation of expression of activating receptors (NKp46) [29], NKp30 [32] and inhibitory receptors (KIR) in humans [33].

Ageing is a primary risk factor for developing neurodegenerative diseases [1, 34] in which prevalence and outcomes are associated with sex (reviewed in [8]), but the effects of sexual dimorphism on age-related immunosenescent changes has not been well characterized. In this study, we aimed to examine sex differences in immunosenescence in aged C57BL/6J mice. We also interrogated the effect of ageing and sex on cytokine profiles in plasma and whole blood. Additionally, we investigated alterations in NK cell phenotypes and their functional capacity with ageing and identified sex-specific changes.

## Results

### Age-dependent changes in spleen size and cellularity in male and female C57BL/6J mice

**Fig. 1:**
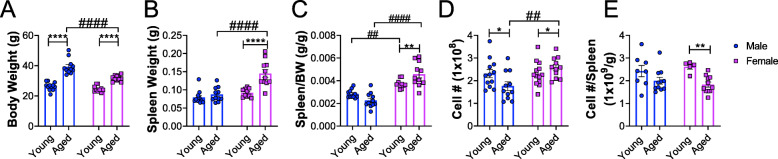
Age- and sex-dependent changes in body and spleen weight. **a** Graph represents body weight (BW) (g). **b** Graph represents spleen weight (g). **c** Graph represents ratio of spleen weight (g) to BW (g). **d** Graph represents total number of splenocytes. **e** Graph represents number of splenocytes per gram of spleen. Males are represented by blue circles and females are represented by pink squares. Young males (*n* = 8–12, 2–3 months); young females (*n *= 5–13, 2–3 months); aged males (*n* = 11, 18–22 months); aged females (*n* = 11, 18–22 months). Data were analyzed by 2-way ANOVA followed by Fisher’s LSD post hoc analysis. Data represent mean ± SEM. Comparisons within sex: *p < 0.05, **p < 0.01, ***p < 0.001, ****p < 0.0001. Comparisons within age: #*p* < 0.05, ##*p* < 0.01, ###*p* < 0.001, ####*p* < 0.0001

To determine the effect of age on body and spleen weights, young and aged male and female C57BL/6J mice were euthanized and the body weights and spleen weights were measured. An age-associated increase in body weight was observed across both sexes (Fig. 1a). Spleen weights were significantly increased in aged female mice compared to young female mice and weighed significantly more than those of aged males (Fig. 1b). However, there was no difference in spleen weights with age in male mice (Fig. 1b). When spleen weights were normalized to body weight, females displayed a significant age-dependent increase in spleen/body weight ratio, but there was no age-related difference in males (Fig. 1c). Also, spleen/body weight ratios in females were significantly higher compared to age-matched male counterparts (Fig. 1c). To determine if the total number splenocytes were higher in females, the number of splenocytes from young and aged mice were counted. We observed an age-associated increase in total number of splenocytes in females, while an age-associated decrease was observed in males (Fig. 1d). However, when the number of splenocytes was normalized to spleen weight, an age-dependent decrease in the number of splenocytes per g of spleen was observed in females (Fig. 1e) but no differences were observed in males (Fig. 1e). Taken together, these results demonstrate age-dependent alterations in spleen size and cellularity mainly in females of this mouse strain.

### Sex- and age-dependent changes in splenic leukocyte populations in C57BL/6J mice

To determine the effect of age on splenic leukocyte composition in both sexes, flow cytometry analysis was performed. We determined the relative percentages and total number of different leukocyte populations (B cells, monocytes, neutrophils, NK cells, CD4 + T cells, and CD8 + T cells) of young and aged mice in both males and females.

**Fig. 2:**
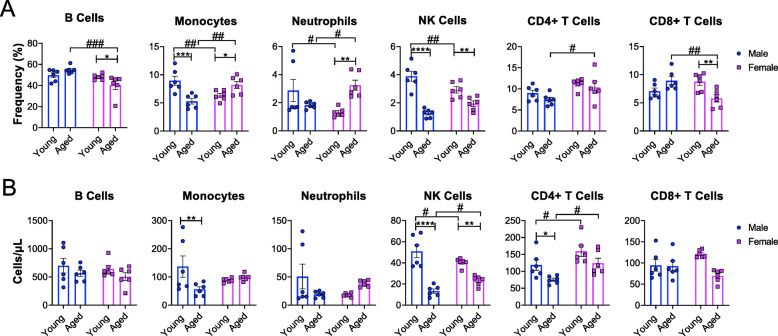
Splenic leukocyte composition is altered in a sex- and age-dependent manner. **a** Plots show frequency of B cells, monocytes, neutrophils, NK cells, CD4 + T cells, and CD8 + T cells in young and aged male and female mice. **b** Plots show total number of B cells, monocytes, neutrophils, NK cells, CD4 + T cells, and CD8 + T cell in young and aged male and female mice. Males are represented by blue circles and females are represented by pink squares. Young males (n = 6, 2–3 months); young females (n = 6, 2–3 months); aged males (*n* = 6, 21–22 months); aged females (*n* = 6, 21–22 months). Data were analyzed by 2-way ANOVA followed by Fisher’s LSD post hoc analysis. Data represent mean ± SEM. Comparisons within sex: *p < 0.05, **p < 0.01, ***p < 0.001, ****p < 0.0001. Comparisons within age: #*p* < 0.05, ##*p* < 0.01, ###*p* < 0.001, ####*p* < 0.0001

We observed aged-associated declines in the relative percentages of monocytes and NK cells in males (Fig. 2a). In females, we observed aged-associated decreases in B cells, NK cells, and CD8 + T cells and increases in monocytes and neutrophils (Fig. 2a). We then compared the differences between the sexes. The relative percentages of B cells and CD8 + T cells were lower in aged females and neutrophils and CD4 + T cells were higher in aged females compared to aged males (Fig. 2a). Interestingly, we also observed sex differences in young mice. The relative percentages of monocytes, neutrophils and NK cells in young females were significantly lower compared to young males (Fig. 2a).

We observed age-associated decreases in the total number of monocytes, NK cells, and CD4 + T cells in males (Fig. 2b), but only the total number of NK cells were reduced with age in females (Fig. 2b). Interestingly, young mice displayed sex differences. NK cell numbers were slightly more in young males and CD4 + T cells were increased in young females (Fig. 2b). Our data demonstrates age-associated changes in splenic leukocyte profiles in both males and females. Importantly, these changes are substantially different in males versus females. Our data implicates that these differences within the secondary lymphoid organs may be associated with sex-biases in age-associated diseases.

### Age-dependent changes in plasma cytokine levels in male and female C57BL/6J mice

**Fig. 3:**
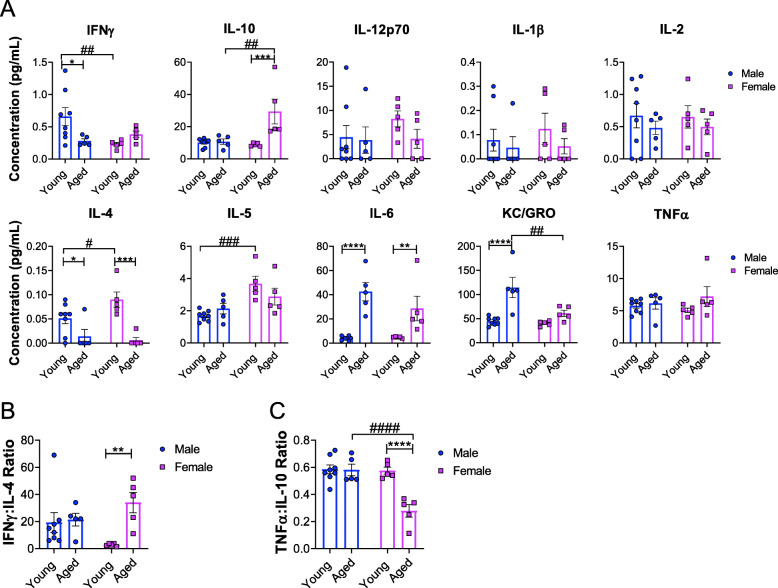
Differential alterations in plasma cytokine content with age and sex. **a** Multiplex pro-inflammatory cytokine/chemokine analysis of plasma samples from young and aged male and female mice. **b** Plots show IFN-γ:IL-4 plasma concentration ratios of young and aged males and females. **c** Plots show TNF-α:IL-10 plasma concentration ratios of young and aged males and females. Young males (*n* = 8, 2–3 months); young females (*n* = 5, 2–3 months); aged males (*n* = 5, 18–19 months); aged females (*n *= 5, 18–19 months). Data were analyzed by 2-way ANOVA followed by Fisher’s LSD post hoc analysis. Data represent mean ± SEM. Comparisons within sex: *p < 0.05, **p < 0.01, ***p < 0.001, ****p < 0.0001. Comparisons within age: #*p* < 0.05, ##*p *< 0.01, ###*p* < 0.001, ####*p* < 0.0001

As we observed sex- and age-specific alterations in immune cell profiles within the spleen, we then analyzed circulating cytokines/chemokines that may influence immune cell populations. To interrogate levels of circulating cytokines/chemokines, a multiplex immunoassay was performed on plasma samples from young and aged mice of both sexes. We observed age-associated decreases in the levels of IFN-γ and interleukin (IL) -4 in plasma and increases of IL-6 and KC/GRO (CXCL1) levels in males (Fig. 3a). We observed an age-associated decrease in the level of IL-4 and increases in IL-10 and IL-6 levels in females (Fig. 3a). Our data also showed differences between sexes at young ages; the level of IFN-γ was significantly lower and IL-4 and IL-5 levels were higher in young females compared to young males (Fig. 3a). The level of IL-10 was significantly higher and the level of KC/GRO was significantly lower in aged females compared to age-matched males (Fig. 3a). No sex- or age-dependent differences in IL-12p70, IL-1β, IL-2, or tumor necrosis factor α (TNF-α) were observed (Fig. 3a). Our data demonstrate age-associated increases in plasma levels of IL-6 and decreases in IL-4 in both sexes suggesting an age-related shift toward a systemic pro-inflammatory state.

To further characterize possible sex- and age-related perturbations in pro- and anti-inflammatory immune responses, the ratio of IFN-γ:IL-4 plasma concentrations to determine Th1/Th2 bias [35] were analyzed. Our data showed an age-dependent increase in the IFN-γ:IL-4 ratio in females but not in males (Fig. 3b) which suggests an age-dependent Th1 bias in females. To further investigate the balance between pro- and anti-inflammatory responses, the ratio of TNF-α:IL-10 plasma concentrations as a measurement of immune homeostasis [36] were calculated. Our data showed an age-dependent decrease in TNF-α:IL-10 ratio in females but no differences were observed in males (Fig. 3c), implicating an age-dependent anti-inflammatory bias in females. Collectively, our data demonstrates dramatic age-associated changes in plasma cytokines in females. Our data illustrates an age-dependent increase in Th1 bias but a decrease in TNF-α:IL-10 in females implicating compensation in overall immune homeostatic status in aged females. This may be indicative of differences between males and females in overall immune function and, therefore, contributing factors for sex-associated diseases.

### Age-dependent changes in whole blood immune response in male and female C57BL/6J mice

**Fig. 4:**
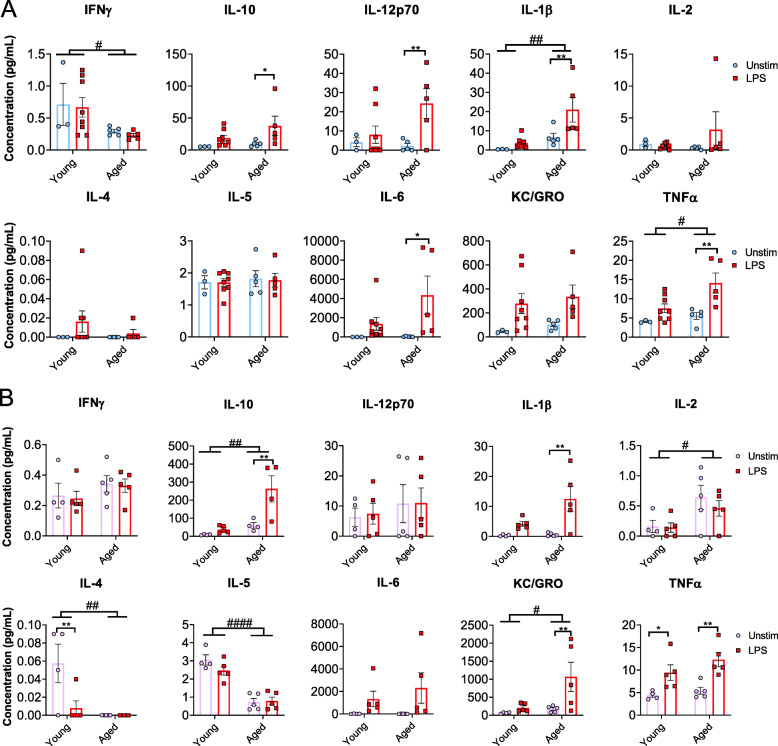
Age- and sex-dependent changes in cytokine/chemokine concentrations following whole blood stimulation. Whole blood samples were collected from young and aged male and female mice. Samples were either unstimulated or stimulated with 10 ng/mL LPS for 24 hrs. **a** Plots represent multiplex pro-inflammatory cytokine analysis of young and aged males following whole blood stimulation. **b** Plots represent multiplex pro-inflammatory cytokine analysis of young and aged females following whole blood stimulation. Young males (n = 3–8, 2–3 months); young females (*n* = 5, 2–3 months); aged males (*n *= 5, 18–19 months); aged females (*n* = 5, 18–19 months). Data were analyzed by 2-way ANOVA with Fisher’s LSD post hoc test. Data represent mean ± SEM. Age: #*p* < 0.05, ##*p* < 0.001, ###*p* < 0.001; Stimulation: *p < 0.05, **p < 0.01, ***p < 0.001

To evaluate age-associated changes in circulating immune cell functionality in male and female C57BL/6J mice, we performed a whole blood stimulation assay. This assay allows for the assessment of overall immune function via cytokine production. Whole blood samples collected from young and aged C57BL/6J mice were stimulated with a relatively low dose of lipopolysaccharide (LPS) (10 ng/mL) to measure the responses in cytokine production of whole blood cells against an inflammatory stimulus. Whole blood cells from aged males displayed significantly increased production of IL-10, IL-12p70, and IL-6 upon LPS stimulation but no changes in the production of IL-2, IL-4, IL-5, or KC/GRO (Fig. 4a). Regardless of stimulation, we observed age-associated increases in IL-1β and TNF-α production and decreases of IFN-γ production in males (Fig. 4a).

In young females, whole blood cells displayed increased TNF-α and decreased IL-4 upon LPS stimulation. Whole blood cells from aged females displayed significantly increased production of IL-10, IL-1β, KC/GRO, and TNF-α upon LPS-stimulation (Fig. 4b). There were no differences observed in IFN-γ, IL-12p70, or IL-6 production in young versus aged females (Fig. 4b). Regardless of stimulation, age-associated increased production of IL-10, IL-2, and KC/GRO and decreased production in IL-4 and IL-5 were observed in females (Fig. 4b).

Collectively, our results suggest a heightened pro-inflammatory response with age in both males and females. Moreover, the age-associated changes in whole blood cell cytokine production against LPS were profound in females which is evidenced by decreases of anti-inflammatory cytokines, including IL-4 and IL-5, in addition to increases of pro-inflammatory cytokines including TNF-α, IL-1β, and KC/GRO.

### Age-dependent changes in splenic NK cell receptor profiles and IFN-γ production in male and female C57BL/6J mice

**Fig. 5:**
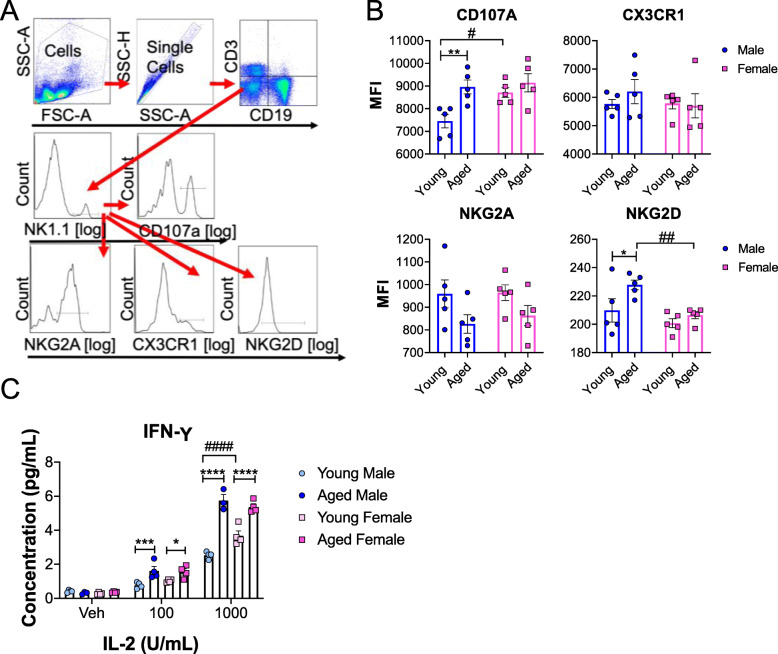
NK cell receptor expression and IFN-γ production are altered in a sex- and age-dependent manner. **a** Gating strategy for flow cytometry data analysis. **b** Graphs represent median florescent intensity (MFI) of receptors on splenic NK cells in young and age males and females. Young males (n = 5, 2–3 months); young females (*n* = 5, 2–3 months); aged males (*n* = 5, 18–19 months); aged females (*n* = 5, 18–19 months). **c** NK cells were isolated from splenocytes of young and aged male and female mice. Cells were then treated with vehicle, 100U/mL IL-2, or 1000U/mL IL-2 for 24 hrs. Supernatant was then collected for IFN-γ ELISA. Graph represents IFN-γ secreted by young and aged male and female NK cells. Young males (*n* = 4, 2–3 months); young females (*n* = 4, 2–3 months); aged males (*n* = 3–4, 18–19 months); aged females (*n* = 4, 18–19 months). Data were analyzed by 2-way ANOVA followed by Fisher’s LSD post hoc analysis. Data represent mean ± SEM. Comparisons within sex: *p < 0.05, **p < 0.01, ***p < 0.001, ****p < 0.0001. Comparisons within age: #*p* < 0.05, ##*p* < 0.01, ###*p *< 0.001, ####*p* < 0.0001

Previously, we have demonstrated that splenic NK cell numbers are increased during the progression of pathology in a preclinical mouse model of Parkinson’s disease (PD) [37] and a potential neuroprotective role of NK cells [38]. Importantly, our data showed a significant age-associated decline in the relative percentages of splenic NK cells in both sexes in Fig. 2. Therefore, further investigation of NK cell phenotype and function was performed. NK cells exert highly specific effector functions through dynamic and combinatorial alterations in expression of various activating and inhibitory receptors. To investigate age-dependent changes in the expressions of NK cell receptors, splenic NK cells from young and aged mice were analyzed via flow cytometry. The relative expression of CD107a (degranulation marker), CX3CR1 (chemotactic receptor), NKG2A (inhibitory receptor), and NKG2D (activating receptor) of NK cells (CD3- CD19- NK1.1+) were analyzed via the gating strategy provided in Fig. 5a. We observed age-related increases in expression of activating NK receptors, CD107a and NKG2D, in males but not in females (Fig. 5b), which implicates that the status of NK cells in aged males are more activated. Interestingly, young females displayed greater expression of CD107a on NK cells compared to young males (Fig. 5b). No additional differences in the expression of NK receptors were observed between young and aged females (Fig. 5b).

IFN-γ is an important immune modulator and is predominantly produced by NK cells [39]. To investigate how IFN-γ production may differ between the sexes and with age we evaluated NK cell IFN-γ production. Splenic NK cells were isolated from young and aged male and female mice and then incubated with various concentrations of IL-2 for 24 hrs. IFN-γ production was measured from the supernatants. We observed dose- and age- dependent responses in IFN-γ production in males and females following IL-2 stimulation (Fig. 5c). Following stimulation with 100U/mL and 1000U/mL IL-2, both aged males and females produced significantly more IFN-γ than their younger counterparts. Interestingly, at 1000U/mL IL-2 stimulation, young females produced significantly more IFN-γ than young males. (Fig. 5c).

Our data demonstrates age-associated increases in splenic NK cell receptor expression in males while IFN-γ production was heightened in both sexes. Our data implicates that these differences in NK cell function between the sexes may be associated with age-associated diseases.

### Aged female NK cells display impaired internalization of alpha-synuclein amyloid fibrils

**Fig. 6:**
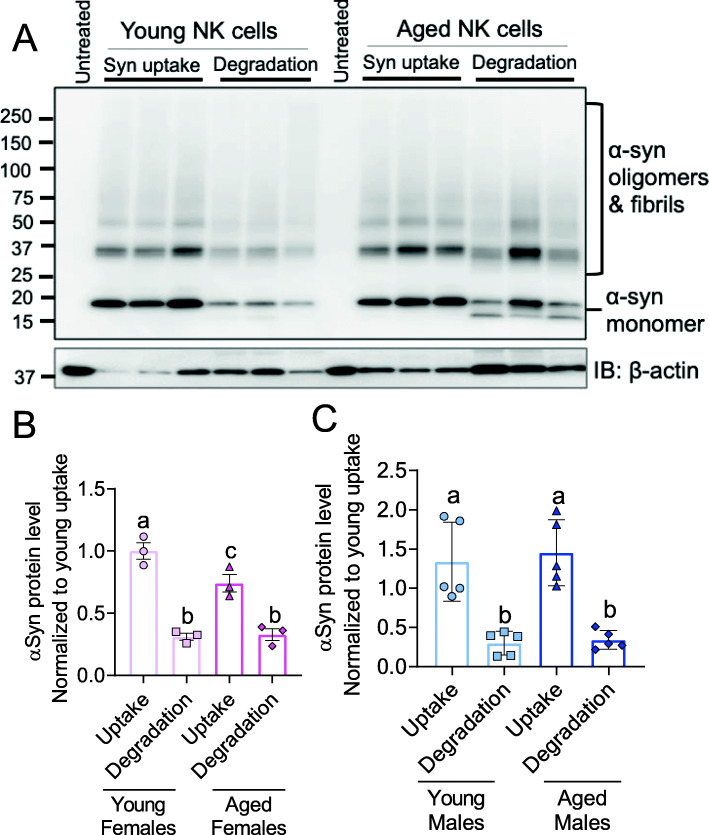
Internalization of α-syn aggregates is impaired with age in females but not males. **a** Western blot analysis of internalized and degraded α-syn aggregates by young and aged female NK cells. NK cells isolated from spleens of young and aged male and female mice were treated with α-syn aggregates for 1 hr (uptake). NK cells were then washed 3 times and incubated in fresh medium for 4 hr (degradation). Untreated samples indicate NK cells that were not treated with α-syn aggregates. **b** Quantification analysis of internalized and degraded α-syn aggregates in females. **c** Quantification analysis of internalized and degraded α-syn aggregates in males. Young males (*n* = 5, 2–3 months); young females (*n* = 3, 2–3 month); aged males (*n* = 5, 18–19 months); aged females (*n *= 3, 21–22 months). Data were analyzed by ANOVA with Tukey post hoc test. Data represent mean ± SEM. *p < 0.05, **p < 0.01, ***p < 0.001

NK cells have been shown to internalize and degrade alpha-synuclein (α-syn) amyloid fibrils [38]. α-Syn is the primary component of Lewy bodies, a pathological hallmark of PD, an age-related neurodegenerative disease. To evaluate age-dependent changes on the NK cell function in clearance of α-syn amyloid fibrils, we evaluated the capacity of NK cells to clear α-syn amyloid fibrils. NK cells were isolated from spleens of young and aged mice, incubated with α-syn amyloid fibrils for 1 hr and the internalization of insoluble α-syn species was analyzed by western blot. In females, our results showed an age-dependent decrease in α-syn internalization (Fig. 6a and b). In males, there is no significant difference in uptake or degradation of α-syn aggregates with age (Fig. 6c). These results implicate that aged female NK cells may have impaired function in resolving α-syn burden, thus contributing to disease pathogenesis.

## Discussion

Cellular senescence is a hallmark of ageing [1]. This process is particularly evident in the immune system and is termed immunosenescence. Immunosenescence and inflammageing, a subset of immunosenescence, lead to system wide alterations in immune cell repertoire and function. While it has been established that the ageing process, including life expectancy and predisposition to disease, may differ between sexes [3], data regarding the underlying physiological alterations and mechanisms for these differences is limited. For example, human female life expectancy tends to surpass that of males [40], but the reason for this inherent difference has yet to be elucidated fully. Here, we sought to explore sexual dimorphism in age-related immune system changes through phenotypic and functional characterization.

It has previously been shown that spleen weight increases with age in male C57BL/6J mice [41], however age-associated changes in female mice have not been well elucidated. Therefore, the total number of splenocytes per gram of tissue was calculated. Our data demonstrated age-related alterations in splenocyte numbers in both sexes and spleen size in females. Alterations in spleen size may be indicative of modifications in the number and distribution of immune cells within the spleen [42]. Additionally, it is known that with age alterations in splenic structure occur, including marginal zone changes and white pulp changes, which may influence splenic immune cell function [42]. Whether the decreased splenic cellularity in females is due to more profound changes in splenic ultrastructure, i.e. white/red pulp redistribution, is unknown.

It has been reported that the number of naïve CD8 + T cells is reduced in ageing mice [43] and humans [44]. Additionally, the ratio of CD4+:CD8 + T cells has been used as a marker of immune activation and an indicator of immunosenescence in humans [45]. Here we determined sex-specific alterations in the composition of immune cells in aged spleen: aged males displayed reductions in monocytes, NK cells, and CD4 + T cells compared to young males and aged females displayed an increase in monocytes and neutrophils and reductions of B cells, NK cells, and CD8 + T cells. The typical CD4+:CD8 + T cell ratio in healthy adults is approximately 2:1 and it has been reported than an inversion in this ratio is related to oxidative stress, chronic inflammation, altered immune system function, and linked to features of immunosenescence [46, 47]. Aged males are more likely to have an inverted CD4+:CD8 + T cell ratio compared to females of the same age [48]. Therefore, changes in the CD4+:CD8 + T cell ratio may have significant implications on response to newly encountered pathogens and inflammatory polarization in a sex-specific manner. Overall, age- and sex-specific alterations in immune cell composition may influence immune system function.

Alterations in immune cell populations may lead to dysregulated immune interactions and effector functions including cytokine production and secretion. Cytokine dysregulations are thought to be involved in immune system remodeling associated with ageing [9]. Therefore, pro-inflammatory related plasma cytokine and chemokine concentrations were also evaluated. Our data showed that plasma levels of IL-6 significantly increased with age in both sexes. In aged females we also observed a significant increase in IL-10 that was not observed in males. Additionally, we observed age-related declines in IL-4 levels in both sexes. It is known that throughout the ageing process IL-6 increases and contributes to inflammageing [9]. Here, we illustrate that aged female mice display a significant increase in IL-6 and a concordant increase in IL-10, a typical anti-inflammatory cytokine, which counteracts the actions of IL-6. Interestingly, it has previously been reported that females do not have a sharp decline in IL-10 concentrations that is observed in males [4, 49]. Thus, aged females may be more adept to counteract pro-inflammatory signals compared to males. T-helper (Th) 1 cells are characterized by the production of IFN-γ and Th2 cells are characterized by the production of IL-4 [50]. Therefore, the ratio of IFN-γ:IL-4 has been used to determine the ratio of Th1 and Th2 cells. We observed an age-related Th1 bias in females as evidenced by the increased IFN-γ:IL-4 ratio. Interestingly, it has been reported previously that age may impact Th1/Th2 bias in male mice [51]. Th2 biased BALB/c male mice take on a more Th1 bias with age [51]. Alterations in Th1/Th2 ratio may influence the type and effectiveness of the immune response. To further characterize alterations in the balance of pro- and anti-inflammatory mediators, the ratio of TNF-α:IL-10 was analyzed. We observed a significant decline in the TNF-α:IL-10 ratio with age in females that was not observed in males. This indicates that with age females may be better equipped to counteract increased inflammation compared to males. The increase of pro-inflammatory and decrease of anti-inflammatory cytokines in aged mice may suggest a shift toward a systemic pro-inflammatory state. A higher basal level of inflammation can have widespread effects including increased susceptibility to viral infections, changes in anti-microbial immunity, and altered ability to modulate inflammation, making ageing adults more susceptible to inflammatory conditions. Additionally, following LPS treatment of whole blood, a significant age-related increase in pro-inflammatory cytokine production in was observed in both males and females. The increases in pro-inflammatory cytokines following stimulation is important in that it may reflect a shift in overall immune functionality with age. Interestingly, we observed significantly decreased concentrations of IL-4 and IL-5 in whole blood samples treated with LPS in aged females. It is important to note that the decreases in cytokine concentrations observed following LPS stimulation are physiologically distinct responses from the decreased cytokine concentrations observed in basal (plasma) conditions. Declines in cytokine concentrations at basal conditions reflect reductions in these cytokines due to age [9, 52]. However, reductions in cytokine concentrations following LPS stimulation may reflect alterations in immune response [53]. Our data suggest a dysregulation in the balance of anti-inflammatory and pro-inflammatory mediators with age in females which may influence overall immune system function.

Of note, we observed that both male and female mice display significant reductions in splenic NK cells with age. Interestingly, human studies have shown that the circulating CD3- CD56 + NK cell population significantly increases with age [30, 33, 54]. Alterations in the frequency of NK cells in the circulatory system and peripheral lymphoid tissues may have immense immunological effects as mounting evidence suggests diverse roles for NK cells including antimicrobial defense [55, 56], clearance of senescent cells [57], modulation of adaptive immunity [58, 59], and resolving inflammation [27, 60]. NK cell immunosenescence may impair crosstalk between the innate and adaptive immune systems [30] which could have substantial implications for the ageing population. Furthermore, it was recently illustrated that NK cells are able to internalize and degrade α-syn aggregates [38], the protein implicated in disease pathogenesis of PD and other age-related synucleinopathies. Therefore, the effects of age and sex on NK cell phenotype and function were evaluated. Our data demonstrated that aged males displayed increased expression of CD107a and NKG2D on NK cells compared to young males. CD107a expression on NK1.1 + cells indicates increased degranulation and cytotoxicity and correlates with both cytokine secretion and NK-cell mediated lysis of target cells [61]. NKG2D is an activating receptor that is constitutively expressed on NK cells and expressed on various cytotoxic cells of the immune system [62]. These findings indicate aged male NK cells may be in an increasingly “primed” state and contribute to the potentiated pro-inflammatory profile we have observed in aged male mice. We also observed an age-dependent hyperresponsive production of IFN-γ upon IL-2 stimulation in both sexes which correlates with the overexpression of CD107a in male NK cells. In humans, aged NK cells have been shown to be hyporesponsive in IFN-γ production upon IL-2 stimulation compared to young NK cells [63]. Alterations in IFN-γ secretion may have impacts on other immune cells as IFN-γ supports Th 1 differentiation, bolsters macrophage function, increases leukocyte migration to sites of infection, and induces upregulation of major histocompatibility complex expression for better T cell recognition of infected or malignant cells (reviewed in [64]). While NK cells are dominant producers of IFN-γ, other sources of IFN-γ include natural killer T (NKT) cells, and Th1 CD4 + and CD8 + T cells [39]. As IFN-γ function is so diverse, the potential implications for dysregulated release of this pro-inflammatory mediator are immense. Many studies have demonstrated a relationship between PD and IFN-γ levels with recent evidence showing elevated blood plasma levels of IFN-γ in PD patients [65].

Since NK cells have been shown to internalize and degrade α-syn aggregates [38], we aimed to interrogate if NK cell function in clearing α-syn aggregates is impaired with ageing. Our results indicate that NK cells from aged female mice have impaired internalization of α-syn but intact degradation compared to those from young female mice. However, there are no age-related differences in the internalization and degradation patterns of α-syn aggregates in NK cells from male mice. The results of this assay do not necessarily correlate with the sex differences observed in human PD (more prevalent in males than females). The combination of reduced NK cell numbers with ageing and impairment of their function could potentially be detrimental, increasing the overall α-syn burden and heightening inflammation both in the periphery and the central nervous system.

## Conclusions

Here, we provide a characterization of immune cell phenotypes and effector functions in young and aged male and female C57BL/6J mice. Aged males and females display different splenic immune cell profiles and circulating cytokine profiles as well as sex-specific alterations in NK cell effector functions. Additionally, this study highlights sex- and age-associated alterations in NK cell numbers and effector functions which implicates their role in age-related diseases such as neurodegenerative disorders.

## Methods

### Animals

C57BL/6J mice (males and females) were obtained from Jackson Laboratory and aged for 2–22 months. Experimental procedures involving the use of animals or animal tissue were performed in accordance with the NIH Guidelines for Animal Care and Use and approved by the Institutional Animal Care and Use Committee at The University of Georgia. Animals were housed in a climate-controlled facility on a 12 hr light/dark cycle with *ad libitum* access to food and water.

### Splenocyte isolation

Spleens were collected from each mouse at 2–3 months or 18–22 months of age. Single cell suspensions were prepared by mechanically homogenizing the spleens and passing through a 70 µm cell strainer (Corning).

### Primary mouse NK cell isolation from spleen

Primary mouse NK cells were isolated from splenocytes using the EasySep™ Mouse NK Cell Isolation Kit according to manufacturer’s directions (StemCell Technologies). Briefly, spleens were isolated from each mouse and a single cell suspension was prepared. Cells were suspended at 1 ⋅ 10^8^ cells/mL. Isolation Cocktail was added to the cell suspension and incubated at RT for 10 min. RapidSpheres™ were added to the splenocyte solution and incubated at RT for 5 min. The cell suspension was then placed into the magnet and incubated at RT for 5 min. The isolated NK cell suspension was then pipetted off and ready to use for experiments.

### Flow cytometry

#### Immune cell profiling

Splenocytes were suspended with FACS buffer (1 mM EDTA, 0.01% sodium azide, 0.1% Bovine Serum Albumin (BSA), 0.02 M phosphate, 0.15 M NaCl, pH 7.2) and then stained for 20 min with anti-FcR/anti-CD16 + CD32/Fc Block (eBioscience) and the following fluorophore-conjugated antibodies for immune cell profiling: anti-CD45-PerCP Cy5.5 (BioLegend), anti-Ly6G-AF700 (Thermo Fisher Scientific), anti-CD19-APC (BioLegend), anti-CD11b-PE (BioLegend), anti-NK1.1-PE Cy7 (BioLegend), anti-TCR-β-Pac Blue (BioLegend), anti-CD4-FITC (BioLegend), and anti-CD8-APC/Cy7 (BioLegend). After staining, cells were washed three times with 200 µl FACS buffer, and then 50 µl of 123count eBeads Counting beads (Thermo Fisher Scientific) were added to allow for quantification of total number of immune cell subtypes following manufacturer’s instructions. Data were acquired on a LSRII instrument (BD Biosciences). Analysis was performed using FlowJo software, version 10.0.8.

#### NK cell receptor profiling

Splenocytes were suspended with FACS buffer (1 mM EDTA, 0.01% sodium azide, 0.1% Bovine Serum Albumin (BSA), 0.02 M phosphate, 0.15 M NaCl, pH 7.2) and then stained for 20 min with anti-FcR/anti-CD16 + CD32/Fc Block (eBioscience) and the following fluorophore-conjugated antibodies for NK cell receptor profiling: anti-CD19-APC (BioLegend), anti-CD3-Pac Blue (BioLegend), anti-NK1.1-PE Cy7 (BioLegend), anti-NKG2A-PE (BioLegend), anti-CD11b-PerCP (BioLegend), anti-CD27-AF700 (BioLegend), anti-CX3CR1-BV510 (BioLegend), anti-CD107a-APC Cy7 (BioLegend), and anti-NKG2D-FITC (BioLegend). After staining, cells were washed three times with 200 µl FACS buffer. Data were acquired on a LSRII instrument (BD Biosciences). Analysis was performed using FlowJo software, version 10.0.8.

### Gating strategies

#### Immune cell profiling

A detailed gating strategy applied to this data can be found in Earls et al., 2019 [37]. Briefly, cells isolated from the spleen were gated first on a forward (FSC) and side scatter (SSC), then total CD45 + leukocytes were gated. This gating strategy allows for the selection of all immune cells while eliminating doublets from analysis. TCR-β + and CD19- T cells were gated from CD45 + parent population. NK 1.1 + NK cells, Ly6G + neutrophils, and CD11b + monocytes were gated from non-B and non-T cells.

#### NK cell receptor profiling

Isolated splenocytes were first gated on forward (FSC) and side scatter (SSC). NK1.1 + NK cells were then gated from the CD3/CD19 double negative population. CD107a+, CX3CR1+, NKG2A+, and NKG2D + NK cells were then gated from NK1.1 + NK cells.

### Preparation of recombinant proteins and aggregates

α-Syn proteins (rPeptide, Bogart, GA, USA) were assembled into aggregates by incubating at 37 °C at a concentration of 1 mg/mL with continuous shaking at 800 rpm for 7 days and aggregation was confirmed by a thioflavin T assay and TEM imaging.

### α-Syn internalization and clearance assays

α-Syn internalization and clearance assays were performed as we previously published [38]. Briefly, primary mouse NK cells were incubated with 5 µg/mL α-syn aggregates (rPeptide) for 1 hr. Cells were then washed 3 times in 1 × PBS. Samples were then either processed for 1 hr internalization or resuspended in fresh medium and returned to the incubator for an additional 4 hr incubation period. Samples were processed for western blot analysis as described in *SDS-PAGE and western blot analysis*.

### SDS-PAGE and western blot analysis

All α-syn internalization and clearance samples were separated into Triton X-100 soluble and insoluble fractions. Cells were lysed in a buffer containing 1% Triton X-100 and 1Xprotease inhibitor mix (Sigma) for 10 min on ice. Lysates were centrifuged at 16,000 g for 5 min at 4 °C. Triton X-100 soluble fraction was then transferred to a new tube and mixed with 4X Laemmli sample buffer. The remaining pellet was then washed with ice cold 1⋅ PBS and centrifuged at 16,000 g for 5 min at 4 °C. The supernatant was then removed and the remaining Triton X-100 insoluble pellet was resuspended in 1X Laemmli sample buffer. Triton X-100 insoluble samples were then sonicated using a high intensity ultrasonic water bath (50% power, 5 sec pulses for 1 min) at 4 °C prior to being loaded on pre-cast 4–20% SDS electrophoresis gels (Bio-Rad, Hercules, CA, USA), transferred onto PDVF membranes (Millipore), and probed with anti-α-syn (MJFR1, Abcam), β-actin (Santa Cruz biotechnology, Santa Cruz, CA, USA) and the appropriate horseradish peroxidase-conjugated secondary antibodies (1:2000; Jackson ImmunoResearch Lab). Immunoreactive bands were visualized with SuperSignal West Femto horseradish peroxidase substrate (Thermo Fisher Scientific, Rockford, IL, USA) according to the manufacturer’s instructions and imaged on a Syngene G:BoxChemi gel documentation station (Frederick, MD, USA).

### Whole blood stimulation assay

Whole blood was collected into EDTA coated tubes by cutting the right atrium. 75–100 µL of blood was plated in duplicates in a round bottom 96-well plate. Once all samples were plated, samples were either unstimulated or stimulated with 10 ng/mL LPS and incubated for 6 hr at 37 °C/5% CO_2_. The plate was then centrifuged at 10,000 rpm for 2 min at RT. Supernatant was then collected for multiplex chemokine and cytokine analysis.

### Primary mouse NK cell culture and IFN-γ ELISA

Primary mouse NK cells were pooled by sex and age and plated at 20,000 cells/well in a round bottom 96-well plate in quadruplicate. Cells were stimulated with vehicle, 100U/mL human IL-2 (PeproTech, cat# 200-02), 1000U/mL human IL-2 and incubated for 24 hrs. Supernatant and IFN-γ levels in NK cell culture media were determined by ELISA (Invitrogen, cat#88-7324-22) according to manufacturer’s instructions.

### Multiplex chemokine and cytokine analysis

Plasma and whole blood stimulation assay samples were analyzed for chemokines and cytokines (IFN-γ, IL-1β, IL-2, IL-4, IL-5, IL-6, KC/GRO (CXCL1), IL-10, IL-12p70, and TNF-α) using a V-PLEX Proinflammatory Panel 1 mouse Kit (cat# K15048D, Meso-Scale Discovery, Rockville, MD) according to the manufacturer’s instructions.

### Statistical analysis

Statistical analyses and graphs were performed and created with Graphpad Prism 8.0 software. Body weight and spleen data, splenic immune cell profile data, plasma cytokine concentration data, whole blood stimulation assay data, NK cell receptor expression data, and IFN-γ production data were analyzed by 2-way ANOVA followed by Fisher’s LSD post hoc test. α-Syn internalization and degradation assays were analyzed by one-way ANOVA followed by Tukey’s post hoc test. Significance was accepted at *p* values < 0.05 and all data are displayed as mean ± standard error of the mean (SEM).

## Data Availability

All data generated or analyzed during this study are included in this published article.

## Abbreviations

α-syn: Alpha-synuclein
ELISA: Enzyme-linked immunosorbent assay
g: Gram
IFN-γ: Interferon-gamma
IL**-**: Interleukin-
KC/GRO: Keratinocyte chemoattractant/human growth-regulated oncogene
LPS: Lipopolysaccharide
MFI: Median fluorescent intensity
NK: Natural Killer
PBMC: Peripheral blood mononuclear cell
PD: Parkinson’s disease
ROS: Reactive oxygen species
RT: Room temperature
SEM: Standard error of the mean
Th: T-helper
TNF-α: Tumor necrosis factor-alpha
